# RACIAL DIFFERENCES IN THE RELATIONSHIPS AMONG VOLUNTEERING, DISCRIMINATION, AND LIFE SATISFACTION

**DOI:** 10.1093/geroni/igae098.3029

**Published:** 2024-12-31

**Authors:** Huei-Wern Shen, Yi Wang

**Affiliations:** University of North Texas, Denton, Texas, United States; University of Iowa, Iowa City, Iowa, United States

## Abstract

Discrimination, whether overt or subtle, has been consistently linked to lower life satisfaction across racial groups. While volunteering has been proven to bring physical and mental health benefits, its potential role in mediating the relationship between discrimination and life satisfaction across different racial groups is unknown. The present study included a subsample of 3115 older adults (65-98) who were non-Hispanic Whites (n=2374), non-Hispanic Blacks (n=449), and Hispanics (n=292) from the 2016 Health and Retirement Study. Discrimination was measured by the six-item Everyday Discrimination Scale (e.g. In your day-to-day life, how often are you treated with less courtesy or respect than other people) and was dummy-coded due to skewness (never experienced vs. ever experienced). Controlling for health-related variables (e.g., self-rated health), SES (e.g., income), contextual features (e.g., urban/rural), and sociodemographics (e.g., gender), findings from stratified multivariate linear regression models showed that non-Hispanic Whites who experienced discrimination reported lower life satisfaction; although older volunteers had higher life satisfaction, volunteering did not mediate the relationship between discrimination and life satisfaction. Among non-Hispanic Black older adults, neither experiencing discrimination nor volunteering was related to life satisfaction. In the Hispanic group, however, experiencing discrimination was related to lower life satisfaction, and volunteering partially mediates the negative impacts of discrimination on life satisfaction. These findings shed light on the possible extended benefits of volunteering in old age. Further research is warranted to better understand the underlying mechanisms through which volunteering may mitigate the negative impact of discrimination on life satisfaction, particularly among Hispanic older adults.

